# Weight-biased attitudes about pediatric patients with obesity in Dutch healthcare professionals from seven different professions

**DOI:** 10.1177/13674935221133953

**Published:** 2023-03-02

**Authors:** Bibian van der Voorn, Roxanna Camfferman, Jacob C Seidell, Rebecca M Puhl, Jutka Halberstadt

**Affiliations:** 1 Department of Health Science, 1190 Vrije Universiteit, Amsterdam, The Netherlands; 2 Rudd Center for Food Policy & Health, Department of Human Development & Family Sciences, University of Connecticut, Hartfort, CT, USA

**Keywords:** weight prejudice, social stigma, health personnel, pediatric obesity, Netherlands

## Abstract

Little is known about the prevalence of negative weight-biased attitudes among Dutch healthcare professionals (HCPs) when treating children and adolescents with obesity and whether interdisciplinary differences are present. Accordingly, we asked Dutch HCPs that treat pediatric patients with obesity to complete a validated 22-item self-report questionnaire about their weight-biased attitudes. In total, 555 HCPs participated from seven different disciplines: 41 general practitioners (GPs), 40 pediatricians, 132 youth healthcare physicians, 223 youth healthcare nurses, 40 physiotherapists, 40 dieticians, and 39 mental health professionals. HCPs from all disciplines reported to experience negative weight-biased attitudes among themselves. Pediatricians and GPs scored highest on negative weight-biased attitudes, including frustrations in treating children with obesity, and feeling less confident and prepared to treat children with obesity. Dieticians scored the least negative weight-biased attitudes. Participants from all groups perceived weight bias expressed by their colleagues, toward children with obesity. These findings are comparable to results reported by adult HCPs from other countries. Interdisciplinary differences were found and underscore the need for more research on contributing factors that impact explicit weight bias among pediatric HCPs.

## Introduction

Weight-related stigma refers to negative attitudes, beliefs, stereotypes or discriminatory behaviors targeted at a person because of their weight (Pearl, 2018). The prevalence of weight discrimination is not only high among the general public, but also among physicians (Sabin et al., 2012). Of concern, many patients with obesity (including children) report experiencing weight stigma from healthcare providers (Palad et al., 2019). For example, in a study of 2449 American adult women with overweight or obesity, 69% of these women reported experiences of weight stigma by their physician. In addition, 46% of these adults reported experiences of weight stigma by nurses, 37% by dieticians and 21% by mental health professionals (Puhl and Brownell, 2006). More recently, multinational evidence shows that as many as two-thirds of adults with high weight report experiencing weight stigma from physicians (Puhl et al., 2021).

In line with these observations, many healthcare professionals (HCPs) acknowledge that they have negative weight-biased attitudes toward patients with obesity, regardless of age (Palad et al., 2019). This includes perceptions that these patients are lazy and lacking willpower or motivation (Rubino et al., 2020). In addition to such explicit weight bias, HCPs also behave implicitly different toward patients with obesity as compared to lower weight patients (Sabin et al., 2012). On average, they spend less time treating patients with obesity, engage in lower quality patient-centered communication, and are less willing to perform diagnostic procedures (Rubino et al., 2020; Phelan et al., 2015). Such attitudes and behaviors, even if unintentional or outside of awareness, contrast with HCPs’ ethical obligations to eliminate their personal biases and combat health inequities (Chen and Anderson, 2021).

Weight-biased care can contribute to psychological and biochemical distress, and adverse health outcomes for adults and children with obesity on the short- and the long-term (Puhl et al., 2016, 2020; Alimoradi et al., 2020; Wu and Berry, 2018; Haqq et al., 2021). Moreover, stigmatization impairs effective prevention and treatment of obesity (Pont et al., 2017). Biased care negatively impacts patients’ motivation, intentions, and commitment to change lifestyle behaviors (Stangl et al., 2019; Palad et al., 2019; Puhl et al., 2016).

Obesity, socioeconomic deprivation, and mental illnesses are among the most strongly stigmatized statuses (Pachankis et al., 2018). With increasing evidence of the globalization of weight bias, weight-related stigmatization is suggested to emerge independently of cultural, social-economical, and contextual factors (Brewis et al., 2018). Moreover internalization of the anti-fat prejudice is suggested to arise already from early childhood (31 months of age) onwards (Ruffman et al., 2016). On one hand, the extent of the problem of the obesity stigma suggests that correlated inequities lie outside of the control of HCPs. On the other hand, policy makers pledge for a redefinition of HCPs’ roles, including their accountability for health equity (Chen and Anderson, 2021).

Insights into HCPs’ obesity stigma offer practical opportunities to make changes that are necessary to improve obesity care toward a stigma-free practice. However, few studies in Europe have compared weight-biased attitudes among different groups of HCPs, and previous work has primarily examined weight-biased attitudes of HCPs treating adult patients (Haqq et al., 2021; Palad et al., 2019). Little is known about weight bias toward youth with obesity in the Dutch healthcare setting.

### Aim

To study the prevalence and interdisciplinary differences of weight-biased attitudes of Dutch HCPs who treat children and adolescents with obesity, including: general practitioners (GPs), youth healthcare physicians (YHCPs), youth healthcare nurses (YHCNs), pediatricians, mental health professionals, dieticians, and physiotherapists.

## Method

### Study population and procedure

HCPs throughout the Netherlands were invited to participate in the present study through their professional associations or public health services. Announcements with a short explanation of the study and research aim were included in their organizations’ newsletter, shown at conferences or shared on LinkedIn. No financial reimbursement was offered to participants and no reminders were sent. Inclusion criteria were sufficient command of the Dutch language and being employed as one of the following professions: GP, YHCP, YHCN, pediatrician, mental health professional, dietician or physiotherapist. There were no exclusion criteria.

The role of pediatricians in the Netherlands is different than in other countries. Dutch pediatricians are medical specialists and provide only secondary or tertiary care, whereas Dutch YHCPs and YHCNs offer preventive care and early intervention support for children (0–19 years).

Professionals gave informed consent for their participation and subsequently completed the questionnaire on paper (when recruited at a conference) or online using Qualtrics.

### Measurements

#### Participant characteristics

The following sociodemographic characteristics were collected through self-report in the questionnaire: gender, age, profession, and years in profession.

#### Weight-biased attitudes

Weight-biased attitudes were measured by a self-report questionnaire developed in prior studies (Puhl et al., 2014a, 2014b), to assess weight bias among HCPs toward patients with obesity, called the “Attitudes of Health Care Providers about Treating Patients with Obesity” scale. This measure has been tested and published previously, using several different samples of HCPs and medical students training in professional health disciplines (Puhl et al., 2014a, 2014b; Phelan et al., 2021). Prior subscale reliabilities ranged from .73 to .90. (Phelan et al., 2021) The questionnaire was translated into Dutch and subsequently translated back into English by an independent translation office. This translation was checked by the developer of the original questionnaire and it was concluded that no apparent differences in the translation were present. In addition, subscales were converted with help of the developer of the original questionnaire.

The “Attitudes of Health Care Providers about Treating Patients with Obesity” questionnaire consisted of 22 items comprising four subscales: 1) Negative attitudes toward patients with obesity (10 items, α = 0.793), 2) Perceived frustrations in treating patients with obesity (6 items, α = 0.798), 3) Perceived confidence and preparedness in treating patients with obesity (2 items, α = 0.741), and 4) Perceived weight bias by colleagues (4 items, α = 0.834). Cronbach’s alphas were determined in the present sample. Participants rated their level of agreement on a 5-point Likert scale ranging from “strongly disagree” to “strongly agree.” For ease of interpretation, 5-point Likert scale ratings were recoded into three ordinal categories: “strongly agree,” “agree,” “neutral,” “strongly disagree,” and “disagree” were recoded into “agreement,” “neutral,” or “disagreement.”

### Statistical analyses

First, the distribution of all continuous baseline and dependent variables was tested. Parametric variables were summarized by mean (SD) and non-parametric variables by median (IQR). Univariate analyses of variance with Bonferroni correction were performed to examine interdisciplinary differences on the parametric subscale scores. In addition, post hoc exploratory analyses were performed to examine the role of gender, age, and number of years in profession. An unpaired t-test was used to analyze the effect of gender and univariate regression analyses were used to analyze the effect of age or number of years in profession, on the subscale scores. A *p*-value <0.050 was considered significant.

## Results

In total, 555 HCPs participated: 41 (7%) GPs, 132 (24%) YHCPs, 223 (40%) YHCNs, 40 (7%) pediatricians, 39 (7%) mental health professionals, 40 (7%) dieticians, and 40 (7%) physiotherapists. Their mean age was 44.4 (SD 11.7) years; 38 (7%) were males and they reported to have mean 18.3 (SD 11.1) years of work experience. A significant age difference existed (*p* = 0.004), with pediatricians being the oldest (mean age 48.0 [SD 9.7] years) and mental health professionals and physiotherapists being the youngest (mean age 39.8 [SD 11.7] and mean age 39.9 [SD 13.0] years, respectively). In addition, a significant difference was found for the variable “years in profession” (*p* < 0.001), with YHCNs having the most experience (mean 20.7 [SD 11.1] years) and mental health professionals having the least experience (mean 12.2 [SD 11.3] years). The male-female ratio was also significantly different between groups of HCPs (*p* < 0.001), with the least number of males in the group of YHCNs (1.4%, *n* = 3) and the largest number of males in the group of GPs (30.8%, *n* = 12).

**Figure 1.**
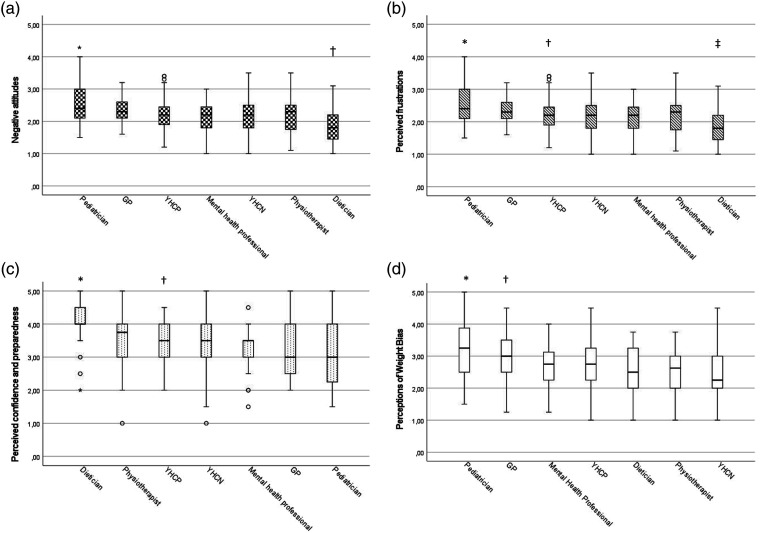
Boxplots of interdisciplinary differences for the four subscales of reported weight-biased attitudes. Per subscale the professions are ranked with the profession that reports the highest mean number of weight-biased attitudes at the far left. Results of post hoc analyses with Bonferroni correction: (a) * Pediatricians reported significant higher negative weight-biased attitudes as compared to all other disciplines, except for GPs. † Dieticians reported significant lower negative weight-biased attitudes as compared to all other disciplines, except for mental health professionals and physiotherapists. (b) * Pediatricians reported significant higher perceived frustrations as compared to all other disciplines, except for GPs and YHCPs. † YHCPs reported significant higher perceived frustrations as compared to YHCNs. ‡ Dieticians reported significant lower perceived frustrations as compared to all other disciplines, except for mental health professionals and physiotherapists. (c) * Dieticians reported significant higher perceived confidence and preparedness as compared to all other disciplines, except for physiotherapists. † Physiotherapists reported significant higher perceived confidence and preparedness as compared to pediatricians and GPs. (d) * Pediatricians reported significant higher perceptions of weight bias by colleagues as compared to all other disciplines, except for GPs. † GPs reported significant higher perceptions of weight bias by colleagues as compared to YHCNs.

Figure 1 and Supplementary Table 1 summarize the weight-biased attitudes across different disciplines. Pediatricians and GPs reported the highest number of negative weight-biased attitudes, including more negative attitudes (Figure 1(a)), more perceived frustrations (Figure 1(b)) and less confidence and preparedness (Figure 1(c)) in treating patients with obesity compared to the other groups of HCPs. Pediatricians and GPs also perceived more weight bias by colleagues compared to other professionals (Figure 1(d) and supplementary Table 1), For example, 60% of the pediatricians and 68% of the GPs had witnessed their colleagues make negative comments about patients with obesity. In contrast, dieticians reported the lowest number of negative weight-biased attitudes, including less negative attitudes, less perceived frustrations, and more confidence and preparedness in treating patients with obesity compared to the other groups of HCPs. However, still 28% of the dieticians reported weight bias by colleagues (Supplementary Table 1).

On a scale-item level, it can be seen that across all groups of professionals, the vast majority reported that patients with obesity should be treated with compassion and respect (ranging between 78% [*n* = 32] agreement for GPs and 95% agreement for dieticians [*n* = 40] or mental health professionals [*n* = 37]) and a substantial number of HPCs feel confident to provide quality care to patients with obesity (ranging between 40% [*n* = 16] agreement for pediatricians and 83% [*n* = 33] agreement for dieticians). However, many physicians (GPs, YHCPs and pediatricians) expressed the view that children with obesity were often non-compliant with treatment recommendations (ranging between 46% [*n* = 19] agreement for GPs and 78% [*n* = 31] agreement for pediatricians), reported that they often felt frustrated with children with obesity (ranging between 33% [*n* = 13] agreement for GPs and 63% [*n* = 25] agreement for pediatricians), and indicated that children with obesity are difficult to deal with (ranging between 27% [*n* = 11] agreement for GPs and 43% [*n* = 17] agreement for pediatricians). Furthermore, 20% of GPs (*n* = 8) and physiotherapists (*n* = 8), and 30% (*n* = 12) of pediatricians would rather treat a non-obese patient than a similar patient with obesity.

No significant effects (*p* > 0.050) were found for participants’ age or number of years in profession on the subscale scores. However, small gender differences appeared: male professionals reported more negative attitudes (mean 2.36 [SD 0.51]) than female professionals (mean 2.16 [SD 0.47]), mean difference 0.196 (95% CI 0.036–0.356), and more perceived weight bias by colleagues (mean 2.93 [SD 0.61]) compared to female professionals (mean 2.61 [SD 0.76]), mean difference 0.328 (95% CI 0.081–0.575).

## Discussion

We aimed to study the prevalence and interdisciplinary differences of weight-biased attitudes of Dutch HCPs who treat children and adolescents with obesity, and found that negative weight-biased attitudes were present across all groups. In addition, weight bias was reported by both HCPs who treat adults and youth, as well as those who only treat youth.

The interdisciplinary differences observed in our study align with findings from a review suggesting that dieticians have slightly less negative attitudes toward people with obesity compared to other HCPs (Jung et al., 2015). Although evidence from previous studies was weak (Jung et al., 2015) and ours is limited by the relatively small sample size per HCP subgroup along with intra-disciplinary differences, we can speculate on possible factors that could explain such differences. It could be that GPs and pediatricians are more negatively influenced by the limited time allocated for weight management than dieticians, which could in turn affect their attitudes and perceived frustrations about treating patients with obesity (Phelan et al., 2015). However, in contrast to physicians’ concerns that it takes a lot of time to address obesity effectively (Auckburally et al., 2021), some evidence suggests that even very brief conversations are sufficient to have an effect (Aveyard et al., 2016). Alternatively, dieticians receive more education in treating patients with obesity compared to GPs and pediatricians. HCPs who understand the underlying mechanisms of obesity and who are educated in a setting where healthcare for patients with obesity is normalized, tend to be less biased and feel more confident in counseling patients with obesity (Phelan et al., 2015; Nestorowicz and Saks, 2021).

In our study, male HCPs reported slightly more negative attitudes and more perceived weight bias by colleagues, as compared to their female counterparts. Similar gender differences have been described in previous studies of healthcare professionals (Sabin et al., 2012; Phelan et al., 2014). Unfortunately, we could not examine other potentially relevant personal characteristics (e.g., BMI of the HCPs) or profession-related factors (e.g., obesity-related training or education) that could affect weight-biased attitudes of HCPs treating youth (Puhl et al., 2014a, 2014b). It will be useful for future research to examine whether these factors help explain potential differences in weight bias amongst different groups of HCPs treating youth, in order to target interventions.

Given previous evidence that weight bias can have adverse consequences on the quality of care and health of patients, including children (Rubino et al., 2020; Haqq et al., 2021), our findings support increasing calls to invest in effective strategies to reduce negative weight-biased attitudes in HCPs (Rubino et al., 2020; Puhl et al., 2020; Pont et al., 2017). Previously, scholars have elaborated on strategies that can help to improve the quality of care by reducing stigmatization barriers for patients with obesity (Phelan et al., 2015; Srivastava et al., 2021), as listed below.

First, offering adequate information and tools to address underlying causes of obesity could reduce negative perceptions and stereotypes about patients with obesity (Mastrocola et al., 2020; Bradbury et al., 2018; Serban et al., 2021). Second, offering interdisciplinary courses to raise awareness about obesity stigma and its harmful impact on health and treatment outcomes is encouraged (Puhl et al., 2020). Third, it is suggested to reduce the focus on body weight as the treatment target. Preferably a combination of clinical characteristics, health indices, and patient-reported outcome measures is used, including quality of life (Ciemins et al., 2021). Fourth, education on patient-centered communication strategies, including motivational interviewing and use of carefully chosen weight-related terminology, is advised to implement as mandatory course in training programs of HCPs (Mikhailovich and Morrison, 2007; Bradbury et al., 2018; Serban et al., 2021; Stuij et al., 2020; Van Maarschalkerweerd et al., 2020; Nnyanzi, 2016). Fifth, it is recommended to promote sensitive and supportive communication about weight-related health among HCPs, including respectful language and neutral weight-related terminology, with people-first language, when talking about weight (Auckburally et al., 2021; Van Maarschalkerweerd et al., 2020; Stuij et al., 2020). Finally, investments are necessary in an infrastructure that offers adequate referral and financial resources for tailored obesity care (Sjunnestrand et al., 2019; Keyworth et al., 2020; Serban et al., 2021).

### Study limitations

Our study is limited by the relatively small sample size per HCP subgroup, making it difficult to draw conclusions for a specific profession. Also, we could not study the potential impact of personal characteristics of HCPs, other than age and sex, or profession-related factors—other than work experience—on their reported weight-bias. In addition, we only assessed self-reported explicit weight bias and did not measure the impact of attitudes on behavior. Furthermore, this was the first study in which these biased ideas were measured cross-sectionally in Dutch HCPs. Therefore, we cannot assess how attitudes might have changed over time.

### Implications for practice

Given the importance of addressing weight bias in pediatric obesity care (Srivastava et al., 2021; Pont et al., 2017; Haqq et al., 2021), our findings underscore the need for more research on the contributing factors that impact explicit weight bias among pediatric healthcare professionals, as well as the need for investments in strategies to reduce stigmatization barriers to improve the quality of care for children with obesity.

## Conclusion

Our findings suggest that negative weight-biased attitudes toward children with obesity exist across all groups of Dutch healthcare providers. Although the relatively small sample size of subgroups limits the ability to draw discipline-specific conclusions, our data point toward a potential interdisciplinary differences with the highest number of negative weight-biased attitudes among pediatricians and general practitioners. Structural investments are necessary to empower these HCPs responsible for managing diagnostics and tailored treatment, to promote high-quality stigma-free obesity care.

## Supplemental Material

Supplemental Material - Weight-biased attitudes about pediatric patients with obesity in Dutch healthcare professionals from seven different professions Click here for additional data file. Supplemental Material for Weight-biased attitudes about pediatric patients with obesity in Dutch healthcare professionals from seven different professions by Bibian van der Voorn, Roxanna, Camfferman, Jacob C Seidell1, Rebecca M Puhl, and Jutka Halberstadt in Journal of Child Health Care
