# Supplementary material for: Weight-biased attitudes about pediatric patients with obesity in Dutch healthcare professionals from seven different professions
Source: J Child Health Care. 2023 Mar 2;27(2):243–52. doi: 10.1177/13674935221133953 (PMC10240630; doi:10.1177/13674935221133953)
Supplement: Supplemental Material - Weight-biased attitudes about pediatric patients with obesity in Dutch healthcare professionals from seven different professions [file sj-pdf-1-chc-10.1177_13674935221133953.pdf]

Supplementary table 1. Weight-Biased Attitudes Across Different Groups of Professionals

	GPs	Pediatricians	YHCP	Mental health professionals	YHCN	Dieticians	Physiotherapists
**Negative attitudes**							
1. I feel that it is important to treat patients with obesity with compassion and respect.	78% (*n*= 32)	85% (*n*= 34)	94% (*n*= 124)	95% (*n*= 37)	94% (*n*= 209)	95% (*n*= 40)	85% (*n*= 34)
2. I feel that patients with obesity are often non---compliant with treatment recommendations.	46% (*n*= 19)	78% (*n*= 31)	49% (*n*= 65)	39% (*n*= 15)	44% (*n*= 99)	15% (*n*= 6)	28% (*n*= 11)
3. I feel that patients with obesity lack motivation to make lifestyle changes.	29% (*n*= 12)	48% (*n*= 19)	26% (*n*= 34)	28% (*n*= 11)	24% (*n*= 54)	7% (*n*= 3)	28% (*n*= 11)
4. Patients with obesity can be difficult to deal with.	27% (*n*= 11)	43% (*n*= 17)	32% (*n*= 42)	15% (*n*= 6)	31% (*n*= 69)	19% (*n*= *8*)	23% (*n*= 9)
5. I would rather treat a non---obese patient than a patient with obesity.	20% (*n*= 8)	30% (*n*= 12)	N/A	0% (*n*= 0)	N/A	3% (*n*= 1)	20% (*n*= 8)
6. I dislike treating patients with obesity.	10% (*n*= 4)	25% (*n*= 10)	N/A	5% (*n*= 2)	N/A	10% (*n*= 4)	10% (*n*= 4)
7. Patients with obesity tend to be lazy.	7% (*n*= 3)	15% (*n*= 6)	2% (*n*= 2)	8% (*n*= 3)	6% (*n*= 13)	5% (*n*= 2)	15% (*n*= 6)
8. Treating a patient with obesity repulses me	0% (*n*= 0)	8% (*n*= 3)	N/A	0% (*n*= 0)	N/A	3% (*n*= 1)	5% (*n*= 2)
9. It is difficult to feel empathy for a patient with obesity.	0% (*n*= 0)	5% (*n*= 2)	1% (*n*= 1)	3% (*n*= 1)	1% (*n*= 3)	2% (*n*= 1)	0% (*n*= 0)
10. I feel disgust when treating an patient with obesity.	2% (*n*= 1)	0% (*n*= 0)	N/A	0% (*n*= 0)	N/A	0% (*n*= 0)	3% (*n*= 1)
**Perceived Frustrations**							
1. Treating patients with obesity is professionally rewarding.	29% (*n*= 12)	28% (*n*= 11)	N/A	51% (*n*= 20)	N/A	78% (*n*= 31)	48% (*n*= 19)
2. I often feel frustrated with patients who have obesity	33% (*n*= 13)	63% (*n*= 25)	34% (*n*= 44)	10% (*n*= 4)	20% (*n*= 45)	7% (*n*= 3)	10% (*n*= 4)
3. Treating a patient with obesity is more frustrating that treating a non---obese patient	22% (*n*= 9)	43% (*n*= 17)	N/A	10% (*n*= 4)	N/A	2% (*n*= 1)	15% (*n*= 6)
4. Treating a patient with obesity is more emotionally draining than treating a non---obese patient	17% (*n*= 7)	18% (*n*= 7)	N/A	11% (*n*= 4)	N/A	10% (*n*= 4)	15% (*n*= 6)
5. Treating a patient with obesity is more stressful than treating a non---obese patient	10% (*n*= 4)	10% (*n*= 4)	N/A	0% (*n*= 0)	N/A	3% (*n*= 1)	10% (*n*= 4)
6. I feel more irritated when I am treating a patient with obesity than a non---obese patient.	7% (*n*= 3)	8% (*n*= 3)	N/A	3% (*n*= 1)	N/A	2% (*n*= 1)	5% (*n*= 2)
**Perceived confidence and preparedness**							
1. I feel confident that I provide quality care to patients with obesity.	42% (*n*= 17)	40% (*n*= 16)	59% (*n*= 78)	67% (*n*= 39)	58% (*n*= 129)	83% (*n*= *33*)	65% (*n*= 26)

2. I feel professionally prepared to effectively treat patients with obesity.	34% (*n*= 14)	40% (*n*= 16)	N/A	26% (*n*= 10)	N/A	90% (*n*= 36)	58% (*n*= 23)
**Perceptions of Weight Bias**							
1. I have heard/witnessed other professionals in my field make negative comments about patients with obesity	68% (*n*= 28)	60% (*n*= 24)	34% (*n*= 45)	46% (*n*= 18)	22% (*n*= 49)	43% (*n*= 17)	28% (*n*= 11)
2. Other health providers in my field often have negative stereotypes toward patients with obesity.	39% (*n*= 16)	53% (*n*= 21)	24% (*n*= 32)	26% (*n*= 10)	14% (*n*= 32)	28% (*n*= 11)	13% (*n*= 5)
3. My colleagues tend to have negative attitudes toward patients with obesity.	15% (*n*= 6)	35% (*n*= 14)	13% (*n*= 17)	21% (*n*= 8)	8% (*n*= 18)	8% (*n*= 3)	8% (*n*= 3)
4. Health care providers feel uncomfortable when caring for patients with obesity.	22% (*n*= 9)	28% (*n*= 11)	11% (*n*= 14)	10% (*n*= 4)	7% (*n*= 16)	5% (*n*= 2)	10% (*n*= 4)

Reported numbers reflect percentage of agreement (*n* within this subgroup). N/A is stated when this specific question is not related to the job description of that discipline.
GP= General Practitioner; YHCP= Youth Health Care Physician; YHCN= Youth Health Care Nurse
